# Financial stress and depression in adults: A systematic review

**DOI:** 10.1371/journal.pone.0264041

**Published:** 2022-02-22

**Authors:** Naijie Guan, Alessandra Guariglia, Patrick Moore, Fangzhou Xu, Hareth Al-Janabi

**Affiliations:** 1 Institute of Applied Health Research, College of Medical and Dental Sciences, University of Birmingham, Edgbaston, Birmingham, United Kingdom; 2 Department of Economics, University of Birmingham, Edgbaston, Birmingham, United Kingdom; Universidade Federal do Rio Grande do Sul, BRAZIL

## Abstract

Financial stress has been proposed as an economic determinant of depression. However, there is little systematic analysis of different dimensions of financial stress and their association with depression. This paper reports a systematic review of 40 observational studies quantifying the relationship between various measures of financial stress and depression outcomes in adults. Most of the reviewed studies show that financial stress is positively associated with depression. A positive association between financial stress and depression is found in both high-income and low-and middle-income countries, but is generally stronger among populations with low income or wealth. In addition to the “social causation” pathway, other pathways such as “psychological stress” and “social selection” can also explain the effects of financial stress on depression. More longitudinal research would be useful to investigate the causal relationship and mechanisms linking different dimensions of financial stress and depression. Furthermore, exploration of effects in subgroups could help target interventions to break the cycle of financial stress and depression.

## Introduction

Depression is one of the most common mental health problems and is marked by sadness, loss of interest or pleasure, feelings of guilt or low self-worth, disturbed sleep or appetite, feelings of tiredness, and poor concentration [1]. Depression is a leading cause of disability and poor health worldwide [1] and is expected to rank first worldwide by 2030 [2]. According to a survey from the World Health Organization, more than 322 million people, which accounted for approximately 4.4% of the world population, suffered from depressive disorders in 2015 [3]. The lifetime risk of developing depression was estimated to be 15%-18% [4]. Mental health problems including depression have imposed a heavy economic burden on individuals and households who are suffering from mental disorders and even on society [5–7]. Specifically, the global costs of mental health problems are increasing each year in every country. Those costs are estimated to reach approximately 16 trillion dollars by 2030 [8, 9]. There is a considerable need to explore the risk factors of mental disorders or the determinants of mental health, which will inform preventive strategies and actions aimed at reducing the risk of getting mental disorders and thereby promoting public mental health.

Many social and economic determinants of depression have been identified. These include proximal factors like unemployment, low socioeconomic status, low education, low income and not being in a relationship and distal factors such as income inequality, structural characteristics of the neighbourhood and so on [10–12]. Research has emerged in the past two decades focusing on the association between the individual or household financial stressors and common mental disorders such as depression and anxiety. However, findings regarding the relationship between different indicators of financial stress and depression are inconclusive in the previous literature. Studies have shown positive associations between depression and various indicators of financial stress such as debt or debt stress, financial hardship, or difficulties [13–15]. Some other studies find no relationships when financial stress was indicated by low income. For example, Zimmerman and Katon [16] found that when other socioeconomic confounders were considered, no relationship between low income and depression was observed. Besides, there is evidence showing a negative association between low income and major depressive disorder in South Korea [17]. A 2010 review on poverty and mental disorders also finds that the association between income and mental disorders (including depression) was still unclear [18].

The social causation theory is one of the theories that has been proposed to explain possible mechanisms underlying the effect of poverty on mental disorders [18, 19]. It states that stressful financial circumstances might lead to the occurrence of new depressive symptoms or maintain previous depression. This might be due to exposure to worse living conditions, malnutrition, unhealthy lifestyle, lower social capital, social isolation, or decreased coping ability with negative life events. Individuals or households with limited financial resources are more vulnerable to stressful life events (e.g., economic crises, public-health crises), which might increase the risk of mental health problems [18–20]. However, practically, social causation might not be applicable to situations where individuals are not in poverty or deprivation but still can experience depression due to financial stress.

Reviews to date have examined the relationship between debt specifically and broader mental health outcomes with depression being one of them. For example, two reviews published in 2013 and 2014 reviewed the literature on the relationship between debt and both mental health and physical health [21, 22]. They concluded that there was a significant relationship between personal unsecured debt or unpaid debt obligations and the increased risk of common mental disorders, suicidal ideation and so on [21, 22]. In terms of depression, they found that there was a strong and consistent positive relationship between debt and depression. Another focus of the literature is on the relationship between poverty and mental health problems including depression in low-and middle-income countries (LMIC). In those reviews, indicators of poverty include low socioeconomic status, low income, unemployment, low levels of education, food insecurity and low social class [18, 23]. Both reviews find a positive relationship between poverty and common mental disorders, which exists in many LMIC societies regardless of their levels of development. Being related to low income, factors such as insecurity, low levels of education, unemployment, and poor housing were found to be strongly associated with mental disorders, while the association between income and mental disorders was unclear.

The reviews discussed above focus mainly on the relationship between debt or poverty and mental health outcomes. As sources of financial stress are complex and multidimensional, indicators such as low income or debt are not the only economic risk factor of mental health problems. Other sources of financial stress such as lack of assets, economic hardship or financial difficulties (e.g., whether an individual finds it difficult to meet standard living needs like buying food, clothes, paying bills and so on) might also relate to depression. In addition, various sources of financial stress might be related to mental health problems in different ways. Based on the existing reviews, it is still unknown which domains of financial stress have clearer associations with depression and whether there is heterogeneity in the relationship between financial stress and depression for different populations and contexts. Moreover, the existing reviews do not discuss the possible mechanisms underpinning the association between financial stress and depression. To better understand the association between financial stress and depression and the possible mechanisms underlying it, a systematic review was conducted bringing together a wide range of indicators of financial stress. The eligible economic indicators of financial stress in this review include objective financial variables like income, assets, wealth, indebtedness; as well as measures that capture subjective perceptions of financial stress, such as perceived financial hardship (e.g., subjective feelings of sufficiency regarding food, clothes, medical care, and housing), subjective financial situation (e.g., individuals’ feelings about their overall financial situation), subjective financial stress, subjective financial position, and financial dissatisfaction.

This study aims at providing a comprehensive review of the association between different financial stressors and depression considering the characteristics of the associations of interest and discussing the proposed mechanisms underlying the associations. An understanding of the relationship between financial stress and depression would not only advance our understanding and knowledge of the economic risk factors of mood disorders but also provide policymakers with more understanding of additional public mental health benefits of intervention aimed at alleviating poverty and/or at improving people’s financial conditions.

## Methods

### Search strategies

A systematic review of published literature was conducted using online searches on bibliographic databases. At the first stage, six bibliographic databases including CINAHL, PsycINFO, EMBASE, EconLit, AMED, and Business Source Premier were searched for related peer-reviewed journal articles to April 2019. The search terms are listed in Table 1. The broad strategy was to combine terms related to finances, with terms related to depression, and terms related to the unit of analysis (individual, household etc). Several key studies that were eligible for inclusion criteria were pre-identified. Before the formal search, pilot searches were performed to make sure the pre-identified key studies can be found by the search. More details of search strategies are displayed in S1 Appendix. All the search results were limited to the English language. No time restriction was added to the search. The reference lists of the eligible studies and several relevant review papers were checked manually to supplement the main electronic searching.

**Table 1:** A simplified search strategy.

Search term sets	Combination strategy
**1) Finances or financial situation**	“income” or “debt*” or “indebt*” or “loan*” or “mortgage” or “wealth” or “asset* or “financ*” or “economic situation*” or “economic stat*” or “economic condition*” or “economic position*” or “economic hardship*” or “economic str*” or “economic difficult*” or “financial situation*” or “financial stat*” or “financial condition*” or “financial position*” or “financial str*” or “financial hardship*” or “financial satisf*” or “financial difficult*” or “poverty” or “deprivation”
**2) Depression**	“depress*” or “mood disorder” or “depressive disorder*” or “depressive symptom*” or “depressed mood*” or “affective disorder*” or “dysthymia*”
**3) Household/individual**	“household*” or “family” or “individual” or “personal”
**Boolean logic**	1) and 2) and 3)

### Eligibility criteria

The inclusion and exclusion criteria for study selection were designed to ensure a focus on primary studies and secondary studies conducted on adults, using measures of financial stress (exposure) and depression (outcome). The eligibility criteria were tested on a selection of papers by multiple members of the study team to ensure that studies were categorised accurately. Based on the piloting process, the eligibility criteria were further modified. The following is the list of the final inclusion and exclusion criteria applied in this study.

Studies were included if they met the following criteria: (1) observational and experimental studies on the relationship between the individual or household financial stress and depression or depressive symptoms; (2) original research in peer-reviewed journals; (3) conducted on general population samples aged 18 and over; (4) used indicators which capture different dimensions of an individual or household financial stress, such as income, assets, debt, wealth, economic hardship, financial strain, financial stress, and financial satisfaction; (5) studies that measure depression through both non-clinical and clinical techniques (e.g., Centre for Epidemiologic Studies Depression Scale), were eligible for this review.

Studies were excluded if they were: (1) systematic reviews, dissertations, conference abstracts, or study protocols; (2) studies focusing on a specific population including female-only, male-only, people with a special occupation (e.g., soldiers), migrants, or people with specific illnesses; (3) studies relating to societal (as opposed individual) economic circumstances (e.g., income inequality measured at the community level or the country level) or shocks to the macroeconomy (e.g., stock market crashes, hyperinflation, banking crises, economic depressions, and financial crisis); (4) studies that only reported the joint association between several socioeconomic determinants and depression. These were included only if the association related to individual or household finances were reported and explained individually; (5) studies based on overall mental health, or other types of mental disorders (e.g., anxiety, suicide, self-harm, bipolar disorder, schizophrenia, dementia) where the association between household financial stress and depression was not reported and explained independently.

### Study selection

**Fig 1:**
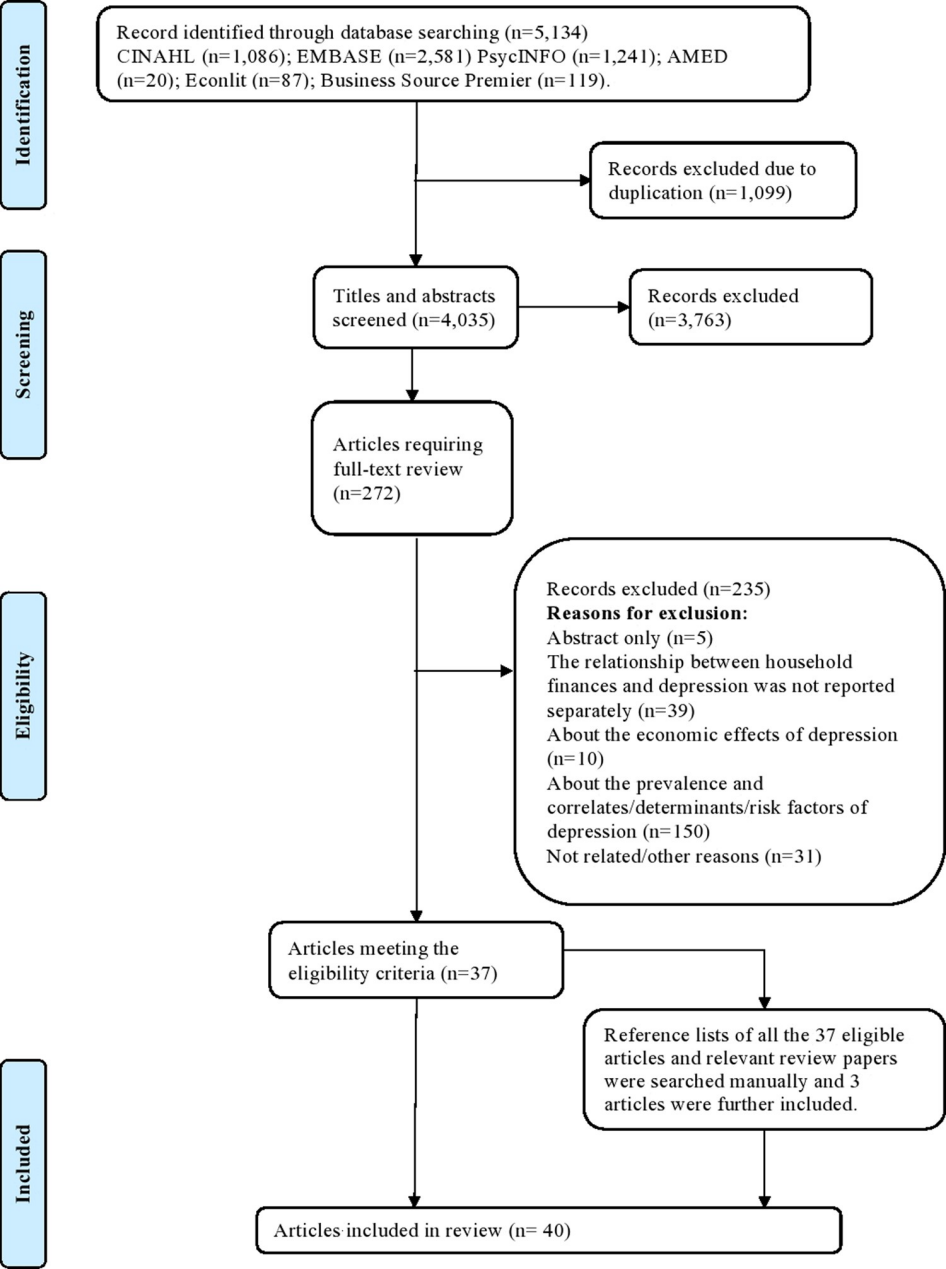
Study selection flow chart.

Search strategies were applied to six databases (i.e., CINAHL, PsycINFO, EMBASE, EconLit, AMED, and Business Source Premier EBSCO) to generate a long list of candidate studies. All the search results were exported into Mendeley. Search results, after the removal of duplicates, were screened for relevance using the title and abstract information. The full texts of relevant articles were then checked for eligibility based on the selection criteria. Screening and selection were undertaken by two reviewers independently. All authors were consulted when a disagreement arose. The study selection process and reasons for study exclusions were recorded in a flow chart shown in Fig 1.

### Data analysis

A pre-designed data collection form (listed in S2 Appendix) was used for the data extraction process. The data extracted from the eligible studies covered the following categories: (1) characteristics of studies: year, author, journal, aim of study, countries, study type, data sources, responsible rate, level of study, eligibility of ethical approval; (2) characteristics of the population: sample size, age group, mean age of the participants, gender; (3) depression measures, definition of depression, validity of the measures; (4) measures of financial stress (exposures) used, measures of the exposure, definition of the exposure, validity of the measures; (5) statistical analysis: econometric methodologies, covariates, whether reverse causality was taken into account, whether there are subgroup analyses and methods (6) main results. The key information being extracted is presented in S1 and S3 Tables, which is a simplified version of the extracted data.

This review focused on the association between each dimension of financial stress and depression and analysed the heterogeneity of the association in different contexts. The eligible studies were reviewed narratively, and the results were stratified by different indicators of financial stress (e.g., low income, low assets, low wealth, debt, financial difficulties and so on). Causal inferences and proposed mechanisms underlying the association between financial stress and depression based on the reviewed evidence were discussed in the discussion section. No meta-analysis was conducted to pool the reviewed evidence due to the substantial heterogeneity in the measurements and definitions of the exposure and outcome variables, study context, and methodologies.

### Quality assessment

The quality of the included studies was assessed using an adapted version of the Quality Assessment Tool for Quantitative Studies used in Glonti et al. [24] (see S3 Appendix). The original version of this tool is developed by the Effective Public Health Practice Project (EPHPP) [25]. Seven key domains relating to study design, selection bias, withdrawals, confounders, data collection, data analysis and reporting were considered. Studies can have between six and seven component ratings. The score of each domain equals 1 if the quality is high, 2 if the quality is moderate and 3 if the quality is low. An overall rating for each study was determined based on the ratings for all domains. The overall rating of studies’ quality was classified as high, moderate, or low. Full details of the design and usage of the quality assessment tool can be found in Glonti et al. [24]. The quality assessment of the included studies was conducted by two reviewers independently. The results of the quality assessment were based on consensus between the two reviewers.

## Results

5,134 papers were identified after searching online databases including CINAHL, PsycINFO, EMBASE, EconLit, AMED, and Business Source Premier. The flow chart for the study selection process is displayed in Fig 1. The total number of papers after removing duplicates was 4,035. Both titles and abstracts of the identified 4,035 papers were screened. 3,763 papers were removed since they did not satisfy the eligibility criteria. The full texts of the remaining 272 papers were accessed and further screened separately by the two reviewers based on the eligible criteria. 235 papers were further excluded leading to 37 studies for consideration. The main reasons for exclusion were that the exposure, the outcome variables of interest, or the targeted population of those studies did not meet the inclusion criteria. Three additional articles were further added after checking the reference lists of all the eligible papers and those of the past relevant review papers [e.g., 18, 21]. 40 articles were finally identified for the data extraction.

### Study characteristics

Regarding the number of reviewed studies by years of publication, most of the reviewed studies were published in the past two decades, with a noticeable spike in the last five years. The majority of studies (32 out of 40) reported evidence from high-income European countries and the USA, Australia, Japan, and South Korea. Eight studies were based on low- and middle-countries including China, Chile, and South Africa. In terms of study design, 17 studies were cross-sectional, and 23 studies were longitudinal. The age groups considered in the 40 studies vary: 17 studies focused on the general adult population including young adults, middle-aged adults, and older adults, while 23 studies focused specifically on working-age, young adults, middle-aged, or older adults. Data of study characteristics were displayed in the data extraction form in S1 Table.

### Measures of depression

The most commonly used measure for depression was the Centre for Epidemiological Studies Depression Scale (CES-D) [e.g., 26–28]. Various versions of the CES-D were used in the reviewed studies: six studies used the full version, that is, the 20-item CES-D; 19 studies used the shortened version of the CES-D scale. Other measures were also used to assess the individual’s depressive symptoms such as the Hospital Anxiety and Depression Scale (HADS) [29], the World Mental Health Composite International Diagnostic Interview (WMH-CIDI) [30, 31], a subsection of the General Health Questionnaire (GHQ depression) [31–33], the Alcohol Use Disorder and Associated Disabilities Interview Schedule-IV (AUDADIS-IV) [34], the 21-item Beck Depression Inventory (BDI) [35] and the Geriatric Depression Scale (GDS) [15, 36, 37]. Two studies used self-reported depression by asking participants whether or not they had any experience of depression [13, 38].

### Measures of financial stress

A wide variety of concepts and measures of financial stress were used across the reviewed studies. The financial exposure in the reviewed studies can be divided roughly into two categories. First, personal or household finances, which include income, assets or wealth, debt or hardship were investigated. These economic indicators were measured in different ways. Some studies measured the total amount of assets while other studies measured assets by counting the number of durable items owned by an individual (such as motor vehicles, bicycles, computers, or cameras) or a household (such as fridges, microwaves, TV, cameras). The measures of debt were more diversified: the onset of debt, the amount of debt in general and of different types of debt, the debt-to-asset ratio, debt problems like over-indebtedness, debt arrears, and debt stress. Financial hardship was defined as difficulties in meeting the basic requirements of daily life due to a lack of financial resources. For example, not having enough money for food, clothes, shelter and medical expenses; being unable to pay bills on time or heat the home; having to sell assets; going without meals; or asking for financial help from others were used by these studies as proxies for financial hardship [30, 39]. Second, some studies examined the associations between depression and subjective perceptions of financial stress such as perceived financial hardship (e.g., subjective feelings of insufficiency regarding food, clothes, medical care, etc.), subjective financial situation (e.g., individual’s feelings of their overall financial situation), subjective financial stress, subjective financial position, financial dissatisfaction and so on.

### Quality of reviewed studies

Full details of the quality assessment of the 40 included studies are displayed in S2 Table and S1 Fig. An observational study design was utilised in all of the included papers. 29 (72.5%), and 11 (27.5%) studies were rated as methodologically strong [6, 13–16, 20, 26–28, 30, 31, 34, 37–53] and moderate [29, 31, 32, 35, 36, 54–59], respectively. Among the 40 included studies, 34 (85%) had a low risk of selection bias, five (12.5%) had a moderate risk and one had a high risk of selection bias. Eight studies were able to be rated on withdrawals and drop-outs: two of them were rated as “strong”, four achieved a “moderate” rating and one received a “weak” rating. We found that 15 studies (37.5%) had a low risk while 25 (62.5%) had a moderate risk of confounding bias. Regarding the data collection, two studies were rated as ‘strong’, 37 received a ‘strong’ score, and one study was rated as ‘weak’. All the studies received a ‘strong’ rating for data analysis except for one study that was rated as ‘weak’. 35 (87.5%) studies received a ‘strong’ rating for reporting, while five studies had a ‘moderate’ quality of reporting.

### Association between income and depression

Eleven studies were identified examining the relationship between individual or household income levels and depression. All controlled for other socioeconomic confounders or/and health status. Seven studies found a statistically significant association between low income and a higher risk of depressive symptoms after adjustment. The positive association between low income and depression was reported in both high-income countries and low- and middle-income countries and found in different age groups (i.e., younger adults, middle-aged adults, and older adults).

The intertemporal relationship between individual or household income and depression was investigated in three longitudinal studies [20, 34, 58]. Osafo et al. found that in the UK, an increase in household relative income (i.e., income rank) was statistically related to a decreased risk of depression at a given time point [58]. The effect of household income at baseline on the risk of showing depressive symptoms in the following time point was weakened but still statistically significant, controlling for the baseline depression level. Lund and Cois reported similar results: they found that lower household income at baseline could predict a worse depression status during the follow-up period in South Africa [20]. Based on evidence from the US, Sareen et al. found that individuals with lower levels of household income faced an increased risk of depression compared to those with higher levels of household income [34]. Furthermore, a reduction in income was also related to an increased risk of depression [34].

Focusing on pension income, which is one of the main sources of household income for the retired population, Chen et al. found that pension enrolment and pension income were significantly associated with a reduction in CESD scores among Chinese older adults, controlling for other socioeconomic factors and health status [43].

The strength of the relationship between income and depression varies and can be affected by how income is measured. For example, compared to absolute income, a household’s relative income level within a reference group was found to be a more consistent household financial predictor of depression [58]. Osafo et al. compared the effect of relative income with that of the absolute value of household income [58]. They found that a deterioration in the rank of household income was associated with a higher possibility of showing depression at a given time point, as well as the subsequent time point [58].

The relationship between income and depression holds for all income groups but is more pronounced among lower-income groups. According to Zimmerman and Katon, the association between depression and income is stronger among people with income levels below the median [16]. Based on a quasi-natural experiment, Reeves et al. also found that the reduction in housing benefits significantly increased the prevalence of depression for low-income UK households [38]. Additionally, the association between pension income and depressive symptoms in older adults were more pronounced among lower-income groups [43]. More broadly, the income-depression relationship might be influenced by the economic status of the regions where households live. For example, Jo et al. found that the association between income and depression was significant among participants from low-economic-status regions, while it was insignificant among participants from high-economic-status regions [55].

### Association between material assets and depression

Two studies on the relationship between assets and depression were identified: one cross-sectional study [29] and one longitudinal study [20]. Those studies showed that assets were a significant predictor of depression after controlling for demographic and other socioeconomic confounders. Furthermore, the household assets-depression association was found to be stronger for individuals with lower levels of assets at baseline [20, 29]. The directions of the assets-depression relationship were investigated in one study. Lund and Cois simultaneously examined both directions of the relationships using a nationally representative survey on South Africa [20]. They found that low levels of individual and household material assets were significantly related to depression in the follow-up period after controlling for age, gender, race and education. Conversely, having more depression symptoms at baseline was significantly associated with lower levels of individual assets in the follow-up period [20].

### Association between wealth and depression

Three studies explored the relationship between wealth and depression in adults. All of them were based on high-income country contexts including the UK and the US and suggested a positive relationship between individual or household low wealth and depression among middle-aged and older adults. Two longitudinal studies examined the association between wealth and depression. Specifically, Pool et al. found that an increase in household wealth was statistically related to a decrease in the risk of depressive symptoms [50]. Osafo et al. compared the effect of relative wealth (i.e., wealth rank) and absolute wealth on depressive symptoms [58]. Their results showed that, instead of the absolute wealth, the wealth rank within a social comparison group was the primary driver of the association between wealth and depressive symptoms [58]. The strength of the relationship between wealth and depression varies according to the level of wealth at baseline [58]. For example, Martikainen et al. found the association between household wealth and depression was most pronounced among the lowest wealth group [33].

### Association between debt and depression

Fourteen studies investigated the association between debt and depression and provided empirical evidence based on high-income countries (Europe and the US) and Chile. Three studies were cross-sectional and all of them reported a positive association between debt (assessed by student debt, the occurrence of any debt, or unsecured debt) and depressive symptoms after controlling for demographic and other socioeconomic factors [6, 27, 53]. Eleven longitudinal studies identified by this review investigated the association between debt and depression over time. The definitions and measures of debt vary across studies. Associations between the occurrence and/or amount of financial debt, the occurrence and/or amount of housing debt, excessive mortgage debt, the occurrence of any debt, the debt-to-asset ratio, and the debt-to-income ratio, on the one hand, and depression, on the other, were investigated in the reviewed studies.

The association between changes in debt status and changes in depressive symptoms was investigated in six studies. Specifically, using five waves of data from the Survey of Health, Ageing and Retirement in Europe (SHARE), Hiilamo and Grundy found that both men and women switched from having no financial debt to having substantial financial debt suffered from a deterioration in depressive symptoms [28]. Also, switching from no mortgage debt to having substantial mortgage debt was positively associated with the deterioration in depressive symptoms among women [28]. Using a large nationally representative dataset from the Chilean Social Protection Survey (SPS), Hojman et al. also found that individuals who were always over-indebted or switch from having moderate levels of debt to over-indebtedness had more depressive symptoms than those who were never over-indebted [46]. Additionally, they found that those who were not over-indebted, regardless of the previous debt status, did not experience a worsening in depression, showing that the effect of over-indebtedness on depressive symptoms faded away as the debt levels decreased [46].

Various measures of debt were used in the reviewed studies such as the occurrence of debt [26, 27, 53], the amount of debt [6, 14, 26, 28, 53], and the debt-to-income ratio or debt-to-asset ratio [14, 46, 48]. The debt-depression relationship varies with different operationalisations of debt with the debt to asset ratio being a more reliable predictor of depression than the total debt. Both Sweet et al. and Hojman et al. found that only the debt-to-assets ratio or debt-to-income ratio (rather than the absolute amount of debt) were consistently and positively associated with higher depression scores before and after adjustment (see S1 and S3 Tables for details of the covariates used) [14, 46].

Different types of debt such as secured debt (e.g., mortgage debt) and unsecured debt (e.g., consumer debt) might be related to the depression in different ways. The reviewed studies reported a positive association between high levels of mortgage debt and high unsecured consumer debt (regardless of the amount) and depression [14, 48, 53]. For example, Leung and Lau examined the causal relationship between mortgage debt and depressive symptoms and found that a high level of mortgage indebtedness (defined as a mortgage loan to home value ratio over 80%) was associated with more depressive symptoms among mortgagors [48]. Both Zurlo et al. and Sweet et al. found that unsecured debt (e.g., consumer debt) was a significant predictor of more depressive symptoms [14, 53]. Three studies compared the effect of different types of household debts on depression [26, 28, 46]. The results of those three studies suggested that the association between household debt and depressive symptoms was predominantly driven by short-term debt. Specifically, unsecured debt (e.g., financial debt), or short-term debt were associated with a higher risk of experiencing depression, while secured debt itself (e.g., mortgage debt) or long-term debt were not related to depressive symptoms. For example, using longitudinal data from the Survey of Health, Ageing and Retirement in Europe (SHARE), Hiilamo and Grundy found that household financial debt was positively and significantly associated with more depressive symptoms, while the effect of household housing debt on depression was weak or even insignificant [28]. Berger et al. found a similar result using longitudinal data from the US. Their results (controlling for baseline characteristics and socioeconomic factors) showed that only short-term debt (i.e., unsecured debt) was positively and statistically significantly associated with depressive symptoms, while the effects of mid-term and long-term debt (e.g., mortgage loan) on depressive symptoms were not significant [26].

However, it is not always the case that the association between debt and depressive symptoms is only driven by consumer debt. As reported in two longitudinal studies by Hiilamo and Grundy and by Gathergood, a secured debt like mortgage might be associated with depression when the secured debt becomes a problem debt [28, 32]. Hojman et al. found that mortgage debt had no association with depressive symptoms, while consumer debt was positively and significantly related to more depressive symptoms [28]. Nevertheless, both Hojman et al. and Alley et al. found that mortgage arrears had a significant effect on more severe depression, even when the effect of consumer debt on depression was controlled [40, 46]. In line with their study, Gathergood also found that housing payment problems were strongly associated with a higher depression score [32].

### Association between financial hardship and depression

The association between financial hardship and depression was reported in four studies, all of which were based on high-income countries such as the US and Australia [15, 30, 39, 52]. They all observed a cross-sectionally positive relationship between financial hardship and depression, which holds after adjustments (see S1 and S3 Tables for details of the covariates used). The intertemporal association between financial hardship and depressive symptoms was reported in two longitudinal studies [15, 39]. However, the consistency of the findings is sensitive to the statistical methods applied. Mirowsky and Ross found that current financial hardship was associated with a subsequent increase in depression in the US [39]. The other study only observed an association between financial hardship at baseline and baseline depression, as well as a weak or even no association between prior financial hardship and current depression [15]. When the same statistical strategy was applied, the findings from Butterworth et al. were consistent with those were observed in Mirowsky and Ross’s study [15, 39].

Furthermore, the reviewed studies showed that the effect of past financial hardship on depressive symptoms decayed with time. In other words, current financial hardship mattered the most for current depressive symptoms. Following Mirowsky and Ross, changes in financial hardship were stratified into four types [39]. An individual experiencing (not experiencing) current financial hardship and hardship in the past belongs to the always hardship group (no hardship group). An individual experiencing only current (past) financial hardship belongs to the new hardship group (resolved hardship group). Mirowsky and Ross found that the effects of consistent hardship and new financial hardship (3 years later) on depressive symptoms were positive and significant [39]. Moreover, there was no significant difference in the follow-up depressive symptoms between the consistent hardship group and the new hardship group [39]. Furthermore, the association between both resolved hardship and no hardship on depressive symptoms was not significant [39]. Consistent with this, Butterworth et al. also found that the individuals who currently experienced financial hardship were more likely to have depression than those who only experienced financial hardship in the past or never experienced it [15].

Age was the most analysed moderator of the association between financial hardship and depressive symptoms among the reviewed studies. This review found that there is no consistency in terms of the association between financial hardship and depression across different age groups. Butterworth et al. reported that the effect of financial hardship on depressive symptoms increased with age among Australian adults [30]. However, Butterworth et al. and Mirowsky and Ross reported different results [15, 39]. Specifically, they found that the positive association between financial hardship and depressive symptoms decreased with age in the US. In contrast to the two studies listed above, Butterworth et al. did not find any statistically significant differences regarding this association among different age cohorts in Australia [15].

### Association between subjective financial strain and depression

Eleven studies examined the association between subjective financial indicators (i.e., subjective financial strain, financial dissatisfaction or financial stress) and depression, providing empirical evidence based on high-income countries (Europe, the US, the UK, Japan and Korea) and on China. All of them (including four cross-sectional and seven longitudinal studies) reported a positive relationship between subjective financial strain and depression, holding after adjustments (see S1 and S3 Tables for details of the covariates used)). The intertemporal association between subjective financial strain and depression was reported in two studies [44, 59]. For example, Richardson et al. found that increased subjective stress at baseline was associated with greater depression over time [59]. Similarly, Chi and Chou also found that higher levels of subjective financial strain measured at baseline were associated with more depressive symptoms after three years among Chinese older people [44]. The association between changes in subjective financial strain and depression was found in one longitudinal study [49]. Using data from the annual Belgian Household Panel Survey, Lorant et al. found that the worsening subjective financial strain was significantly associated with the increased risk of depressive symptoms and that of caseness of depression [49].

The positive and significant association between perceived financial strain in childhood and depression in adults was found in both a cross-sectional study and a longitudinal study [42, 47]. Using cross-sectional data from 19 European countries in 2014, Boe et al. found that younger adults (25–40) who had experienced financial difficulties as children had higher depression scores in adulthood, while older adults (over 40) did not [42]. A similar association between adverse childhood financial situation and adults’ depression was also found in a longitudinal study [47]. Based on a national representative sample of 9,645 South Korean adults without depressive symptoms at baseline, Kim, et al. found that experiencing financial difficulties in childhood was associated with the increased chance of depression in adulthood [47]. Furthermore, the effect of experiencing financial difficulties in childhood on depression was weaker than that of current financial difficulties [47].

The gender difference of the association between perceived financial strain and depression was examined in two studies and no statistical difference between females and males was observed, though women tended to report worse depression [36, 44].

## Discussion

### Summary and discussion of the findings

This systematic review is the most comprehensive synthesis of observational studies quantifying the association between indicators of financial stress and depression in both high- and low- and middle-income countries to date. Findings regarding the relationship between financial stress and depression vary across different indicators of financial stress. Economic indicators such as material assets, unsecured debt, financial hardship, and subjective measures of financial stress are relatively strong and persistent predictors of depressive symptoms, while absolute income and wealth levels have an inconclusive association with depression. The only longitudinal evidence on relative income and relative wealth suggests a stronger relationship between relative income or relative wealth and depressive symptoms than that between absolute income or wealth and depression. Additionally, this review finds that the association between indicators such as income, material assets or wealth and depression is more pronounced in lower socioeconomic groups (i.e., low income or low wealth group). This review is unable to make a conclusion regarding the association between debt and depression across different socioeconomic subgroups. The only evidence is provided in one study showing that there is no difference in the association between debt and depression by assets level. Additionally, there is insufficient evidence to conclude a common pattern regarding the association between financial stressors and depression by gender or age groups, though differences of this relationship across age or gender groups are observed in some of the reviewed studies.

The income-depression association is inconclusive, although income is one of the most commonly used indicators of the individual or household’s economic situation. The reviewed studies consistently reported a positive association between low income and depressive symptoms in univariable analyses. However, this association was largely reduced or even became insignificant when other social and economic factors (such as educational level, employment status and so on) and health status were controlled for [31, 33]. The findings are consistent with the results from the previous reviews and empirical research where different mental disorders were considered including depression [18, 23, 60]. It is likely that income has a close correlation with other dimensions of the socioeconomic condition such as educational levels and employment status that affect an individual’s mental health independently from income *per se* [23].

Furthermore, this review finds that compared to *absolute* income (or wealth), *relative* income (or wealth) in a reference group is a more important risk factor of depression. There is evidence showing a positive association between low-income ranks and current depression scores as well as follow-up depression scores, while no association is found between absolute low income and depression [58]. The findings here are in line with the previous review, which mainly focused on the association between income inequality and depression [61]. Patel et al.’s review concluded that a higher level of income inequality at the neighbourhood level was strongly associated with a higher risk of depression [61]. This review only identified one study investigating the association between relative income or relative wealth and depression. The insufficient evidence on this topic suggests the need for more research to investigate the mental health effects of relative income (or wealth).

Some of the reviewed studies have suggested a positive association between debt and depression despite the substantial heterogeneity in definitions and measurements of debt, study methods, study contexts, and targeted population. The association between debt and depressive symptoms is mainly driven by unsecured debt (e.g., credit card) or late mortgage payments. Secured debt (e.g., mortgage debt) *per se* is not associated with depressive symptoms. However, depression may still be more likely when individuals or households are no longer able to manage their debt or perform debt obligations. For example, the reviewed evidence shows that mortgage arrears have a significant effect on more severe depression, even when the effect of consumer debt and mortgage debt on depressive symptoms are considered within the same model [46, 48]. The findings regarding the relationship between debt and depression are consistent with the findings from the previous reviews on the association between debt and health where depression was one of the outcomes [22, 62].

An important consideration regarding the debt-depression relationship is that having personal or household debt does not always lead to depression, as debt is not always a sign of financial problems. Some personal and household loans are taken to finance housing purchases, business, and investments, which are granted based on the borrower’s financial situation and payback abilities. Additionally, except for stress, debt might also bring benefits to mental well-being by generating consumption, feelings of attainment or satisfaction or making investments [63, 64]. As a result, the financial stress derived from debt could be partially offset by such positive mental well-being effects. The review suggests that future longitudinal research on the impact of debt on depression should consider mediators to understand the nature of the causal association between debt and mental health.

It should be noted that nearly a half of the reviewed studies are cross-sectional, limiting the ability to draw a conclusion on the directions and the causality of the associations between some indicators of financial stress and depression. A few of the longitudinal studies considered the reverse relationship and/or the unobserved bias using econometric methods. The majority of these longitudinal studies mainly focused on the relationship between debt or subjective measures of financial stress and depression. They provide supportive evidence that, debt and subjective financial stress might lead to subsequent depressive symptoms. Longitudinal evidence remains limited as to the understanding of both directions and causality of the relationships between other indicators of financial stress and depression. For example, only three longitudinal studies provided an exploration of the association between income and depression. The casual relationship between some indicators of financial stress (such as low income, material assets, wealth, financial hardship) and depression should therefore be interpreted with caution.

This review includes a number of studies focusing on the older-aged population. The signs of the relationship between financial stress and depression in different age subgroups do not show a significant difference. Despite this, it should be noted that there might be heterogeneity in the magnitude of the relationship between financial stress and depression across different age subgroups. However, it is difficult to identify if including the studies based on adults aged 50 and over would make the generalisability of the findings towards this population. Because a cross-study comparison is almost impossible as there is a substantial heterogeneity in different studies regarding country contexts, measurements of exposures and outcome variables, study methods and so on.

### Mechanisms

Based on the reviewed evidence, three possible mechanisms may be behind the relationship between financial stress and depression.

#### Social causation

Firstly, as highlighted in the introduction, the effects of financial stress on depression can be explained by social causation theory. The reviewed evidence supports the social causation pathway according to which individuals or households who have low income or low wealth are more likely to be exposed to economic uncertainty, unhealthy lifestyle, worse living environment, deprivation, malnutrition, decreased social capital and so on [20, 47, 65]. Those factors might lead to a higher risk of developing depressive symptoms. Individuals or households with limited financial resources are more vulnerable to stressful financial events, which might increase the risk of experiencing depression. This mechanism is applicable to the studies where financial stress is measured by economic indicators related to poverty, such as income poverty, deprivation, and financial hardship.

#### Psychological stress

The reviewed studies also show that subjective measures of financial stress have adverse effects on depression. Indeed, some studies state that subjective financial stress is more important than objective measures such as the amount of debt [13, 41, 66]. Objective indicators of financial stress might have an indirect effect on depression, which is mediated by the individual’s perception of those objective indicators as resulting in financial stress. Experiencing a similar objective financial situation, people may report different perceptions of the objective financial situation due to the heterogeneity of personal experiences, abilities to manage financial resources, aspirations, and perceived sufficiency of financial resources [67]. For example, individuals with limited financial resources are more likely to be concerned about the uncertainty of the future financial situation. The expectation of financial stress, not just their occurrence, may also cause depression. Furthermore, people living in poverty face substantial uncertainty and income volatility. The long-run exposure to stress from coping with this volatility may also threaten mental health [68]. Therefore, it is reasonable to believe that both the respondent’s perception of financial stress and objective measures of financial stress lie at the heart of the relationship between financial stress and depression.

#### Social selection

Other studies suggest that depression might negatively impact the finances of individuals [20, 69]. Social selection theory states that individuals who have mental disorders are more likely to drift into or maintain a worse financial situation [20]. Evidence shows that mental problems might increase expenditure on healthcare, reduce productivity, and lead to unemployment, as well as be associated with social stigma, all of which are related to lower levels of income [18, 65, 70]. However, some scholars argue that the relative importance of social causation and social selection varies by diagnosis [71]. Social causation theory is more important to the relationship between financial stress and depression or substance use; while social selection theory is more important in relation to severe mental disorders such as schizophrenia [70, 72].

### Limitations of the review

This systematic review is the first to comprehensively pool observational studies on the association between individual or household finances and depression or depressive symptoms. However, this review is subject to several limitations. First, since there is substantial heterogeneity in the measurements and definitions of exposure (financial stress) and the outcome variable (depression), targeted populations, and methodologies between studies, a meta-analysis combining the data from the reviewed studies is neither appropriate nor practical. As such, only a narrative approach is used in this review without quantitatively synthesising the data from the studies, which are difficult to compare. Second, the majority of studies reported evidence on high-income countries like the US, the UK, European countries. Therefore, the conclusions of this review are more immediately generalisable to these contexts as opposed to low-and middle-income country contexts. Third, this review undertakes the search on six databases for any related peer-reviewed journal articles without searching for other resources to find grey or unpublished literature and conference abstracts. Excluding the unpublished studies might limit the findings of this review since studies with significant results are more likely to get published [73]. The published studies may lead this review to overestimate the associations between any financial exposures and depressive symptoms. Fourth, the included exposures in this review are the most direct indicators (i.e., proximal indicators) of financial stress. The findings in this review might not be generalisable to the relationship between a distal factor (e.g., job loss) and depression. Evidence has suggested that a significant life event or experience, for example, job loss, is associated with financial stress and thereby can predict subsequent major depression [74]. Future review on financial stress and mental health outcomes might benefit from further including the effect of distal factors (such as job loss, changes in working hours, changes in marital status, and so on) on mental health.

### Implications

This review has a number of implications for public policy around financial circumstances and depression. Firstly, it highlights the role that measures aimed at alleviating financial poverty and inequality could have in improving public mental health. Secondly, it suggests attention needs to be focused on unsecured debt as a public health measure. For example, providing financial counselling services and financial education to those who have debt stress and depression may help them to effectively deal with individual debt problems and associated depression. Meanwhile, the regulation of unsecured debt markets is crucial to the sustainable development of unsecured lending markets, and thereby supports both financial health and mental health. Thirdly, this review highlights the importance of targeted interventions to break the cycle of financial stress and depression. For example, instead of a one-for-all intervention focusing on the general population, interventions targeted at lower socioeconomic groups might be more effective since the association between financial stress and depression is more pronounced in these groups.

In terms of practical support, the interdisciplinary collaboration of psychologists and financial professionals in the development of interventions aiming to break the vicious cycle of depression and financial stress could be useful. For example, interventions such as poverty alleviation programs, the provision of financial advice or financial education might have a beneficial influence on mental health. The collaboration of policymakers in both mental health areas and financial areas might create win-win situations, having mutual benefits for both areas and saving costs to society in the long run.

Finally, this review highlights the need for further research in certain areas. First, this review suggests that more longitudinal research or randomised control trials (where feasible) are needed to further clarify the directions of the causal relationships and possible mechanisms between different financial stressors and depression or other mental disorders. A better understanding of this can help to design more effective interventions either aimed at alleviating financial stress or at improving mental health. For example, anti-poverty programs such as direct cash transfers might be more helpful for families in poverty or deprivation, where social causation plays the main role in the effect of financial stress on depression. However, if financial stress is due to social comparison, rather than absolute poverty, challenging social attitudes may be more beneficial. This review also calls for more future research to investigate the heterogeneity of the relationship and the difference in the direction of the relationship between financial stress and depression or other mental disorders across different populations. This would provide more precise and solid evidence for developing targeted interventions as noted above.

Second, this review finds that the majority of the existing studies on the financial stress-depression relationship are based on high-income countries. However, low-and middle-income countries have higher levels of poverty and economic inequality, as well as a high economic burden caused by mental disorders and low levels of investment in mental health [69, 75]. Therefore, more future research that is based on low-and middle-income country contexts would be important.

## Conclusion

In conclusion, this systematic review of the link between financial stress and depression in adults found that financial stress is positively associated with depression, in particular among low socioeconomic groups. The findings suggest directions for policymakers and the need for greater collaboration between psychology and financial professionals, which will be beneficial to developing targeted interventions either to mitigate depression or alleviate financial stress. Further longitudinal research would be useful to investigate the causality and mechanisms of the relationship between different dimensions of financial stress and depression.

## Supporting information

S1 Checklist PRISMA checklist. (DOC)

S1 Fig Quality assessment of the reviewed studies. (DOCX)

S1 Table Basic characteristics data extraction. (DOCX)

S2 Table Quality assessment form. (DOCX)

S3 Table Methods and findings extraction form. (DOCX)

S1 Appendix Sample search in PsycINFO. (DOCX)

S2 Appendix Data extraction form. (DOCX)

S3 Appendix Quality assessment tool following Glonti et al. (2015) [24]. (DOCX)
