# Supplementary material for: Financial stress and depression in adults: A systematic review
Source: PLoS One. 2022 Feb 22;17(2):e0264041. doi: 10.1371/journal.pone.0264041 (PMC8863240; doi:10.1371/journal.pone.0264041)
Supplement: S1 Fig — (DOCX) [file pone.0264041.s002.docx]

**S1 Fig. Quality assessment of the reviewed studies**
