# Supplementary material for: Financial stress and depression in adults: A systematic review
Source: PLoS One. 2022 Feb 22;17(2):e0264041. doi: 10.1371/journal.pone.0264041 (PMC8863240; doi:10.1371/journal.pone.0264041)
Supplement: S1 Table — (DOCX) [file pone.0264041.s003.docx]

**S1 Table. Basic characteristics data extraction**

| **Study ID** | | | **Study type** | **Level of Study (national level or city level or town level or area level or others)** | | **Years** | | | **Countries** | | **Data source** | | | **Population** | | | | **Exposures** | | **Depression measure** | |  |  |
| --- | --- | --- | --- | --- | --- | --- | --- | --- | --- | --- | --- | --- | --- | --- | --- | --- | --- | --- | --- | --- | --- | --- | --- |
| Asebedo and Wilmarth, 2017 [41] | | | Longitudinal | National representative | | 2012, 2014 | | | USA | | The HRS | | | 8,366 observations, aged 50 and over | | | | Financial strain and financial stress | | CESD-8 | |  |  |
| Alley et al., 2011 [40] | | | Longitudinal | National representative | | 2006-2008 | | | USA | | The HRS | | | ﻿2474 individuals, aged 50 and over | | | | Mortgage delinquency | | CESD-8 | |  |  |
| Berger et al., 2016 [26] | | | Longitudinal | National representative | | 1987/1989, 1992/1994 | | | USA | | The NSFH | | | 8,457–8,516 individuals per dataset, aged between 21 and 65 years of age | | | | Debt occurrence and debt amount; financial debt occurrence and financial debt amount; housing debt occurrence and housing debt amount | | CESD-12 | |  |  |
| Boe et al, 2017 [42] | | | Cross-sectional | International level | | 2014 | | | Europe | | The ESS, round 7. | | | 18,401 respondents, aged 25–75 | | | | Financial difficulties in childhood | | CESD-8 | |  |  |
| Boey and Chiu, 2005 [36] | | | Cross-sectional | Regional level | | 1997/1998 | | | China | | A primary study conducted in Hong Kong | | | 1,034 individuals, aged 70 and over | | | | Financial status: income and perceived income sufficiency | | GDS-15 | |  |  |
| Bridges and Disney, 2010 [13] | | | Longitudinal | National representative | | 1999-2005 | | | UK | | The FACS | | | 1999 sample: 4,549 sample and 2001 sample: 8,062 sample, aged 16 and over | | | | Objective debt variables, subjective debt problem, subjective financial stress | | Self-reported depression | |  |  |
| Butterworth et al., 2009 [15] | | | Longitudinal | Community level | | 2003/2005 | | | South-east Australia | | The PATH Through Life Study | | | 6,715 persons, aged 24-68 | | | | Financial hardship in wave 2, and subjective financial difficulty in wave 1 | | GDS-9 | |  |  |
| Butterworth et al., 2012 [30] | | | Cross-sectional | National representative | | 2007 | | | Australian | | The NSMHWB | | | 8,840 respondents, aged among 16 and 85 | | | | Household income and financial hardship | | ICD-10 | |  |  |
| **Study ID** | | | **Study type** | | **Level of Study (national level or city level or town level or area level or others)** | **Years** | | **Countries** | | | **Data source** | | **Population** | | | **Exposures** | | | | | | **Depression measure** | |
| Chen et al., 2016 [43] | | | Cross-sectional | | National representative | 2012 | | China | | | The CFPS | | 8,636 rural registered individuals in China, aged above 45 years. | | | Pension enrolment and pension income | | | | | | CESD-20 | |
| Cheung and Chou, 2017 [37] | | | Cross-sectional | | City level | 2015 | | China | | | A cross-sectional survey of older persons in Hong Kong | | 1,959 persons aged 65 or over. | | | Income poverty and material deprivation | | | | | | GDS-15 | |
| Chi and Chou, 2000 [44] | | | Longitudinal | | City level | 1992-1995 | | China | | | A two-wave longitudinal survey of older persons in Hong Kong | | 554 older persons aged 70 and over at baseline. | | | Financial strain | | | | | | CESD-20 | |
| Drentea and Reynolds, 2012 [54] | | | Longitudinal | | County level | 2000/2001-2004/2006 | | USA | | | The Miami Disability study | | 1,463 Miami-Dade County residents of adults | | | Occurrence of debt, credit card debt, and debt stress | | | | | | CESD-20 | |
| Drentea and Reynolds, 2015 [45] | | | Longitudinal | | County level | 2000/2001-2004/2006 | | USA | | | The Miami Disability study | | 1,463 Miami-Dade County residents of adults | | | Occurrence of debt and economic hardship | | | | | | CESD-20 | |
| Ellaway et al., 2016 [29] | | | Repeated Cross-sectional | | Regional level | 1997 and 2010 | | UK | | | The THAW 2010 | | 2,092 individuals aged 17 to 95 years. | | | Material assets | | | | | | HADS depression. | |
| Gathergood and John, 2012 [32] | | | Longitudinal | | National representative | From 1991 to 2008 (18 waves) | | UK | | | The BHPS | | 10,000 individuals aged 16 and over. | | | The occurrence of problem debt and debt stress | | | | | | GHQ-12 depression | |
| Gillen et al., 2017 [27] | | | Cross-sectional | | National representative | 2006 | | USA | | | The HRS | | 5,383 individuals aged 51 years and over | | | Debt occurrence, financial assistance from family, public assistance | | | | | | CESD-8 | |
| Hiilamo and Grundy, 2018 [28] | | | Longitudinal | | National representative | 2003/2004, 2006/2007, 2011, 2013, 2015 | | Belgium, France, Germany | | | The SHARE | | 31,409 individuals aged 50 and over | | | Financial debt amount and housing debt amount | | | | | | CESD-12 | |
| **Study ID** | | **Study type** | | | **Level of Study (national level or city level or town level or area level or others)** | **Years** | | | **Countries** | | | **Data source** | | **Population** | | | | **Exposures** | | **Depression measure** | | | |
| Hojman, 2016 [46] | | Longitudinal | | | National representative | 2002, 2004, 2006, 2009 | | | Chilean | | | The SPS | | 14,463 individuals.  Over 18 years old. | | | Financial service ratio, mortgage arrears (MA), and over-indebtedness (OI) | | | CESD-8 | | | |
| Jo et al., 2011 [55] | | Cross-sectional | | | Regional level | 2006/2007 | | | Korea | | | A survey conducted from November 2006 to November 2007 in two regions located in Seoul. | | 966 and 992 in the A ‘Gu’ and B ‘Gu’, respectively, Adults. | | | | Economic status | | CESD-20 | | | |
| Kim et al., 2016 [47] | | Longitudinal | | | National representative | 2010 to 2013 | | | Korea | | | The KOWEPS | | 9,645 individuals without depressive symptoms from South Korea, aged 20 and above. | | | | Childhood economic status, current economic status (household income) | | CESD-11 | | | |
| Krause et al., 1991 [56] | | Cross-sectional | | | Both are national representative | USA: 1986; JAPAN: 1987 | | | USA, Japan | | | The US. Data: the Americans' Changing Lives Survey; Japanese Data: a random-probability survey conducted in Japan. | | US: 1,523 individuals; Japan: 1,517 individuals; older adults aged 60 and over | | | | Financial strain | | CESD-6 | | | |
| Krause et al., 1998 [57] | | Cross-sectional | | | City level | 1991 | | | China | | | 1991 Survey of Health and Living Conditions of the Aged in Wuhan City. | | 2,749 individuals, aged 60 and over | | | | Financial strain, received economic support, anticipated economic support. | | CESD-6 | | | |
| **Study ID** | | **Study type** | | | **Level of Study (national level or city level or town level or area level or others)** | **Years** | | | **Countries** | | | **Data source** | | **Population** | | | | **Exposures** | | **Depression measure** | | | |
| Leung and Lau, 2017 [48] | | Longitudinal | | | National representative | From 1992 to 2008 (9 waves) | | | USA | | | The HRS | | 30,548 individuals, aged 50 and over | | | | Mortgage loan to house value | | CESD-8 | | | |
| Lorant et al., 2007 [49] | | Longitudinal | | | Regional level | From 1992 to 1999 (8 waves) | | | Belgian | | | The Belgian Household Panel Survey | | 11,909 individuals aged 16 and over | | | | Subjective financial strain, poverty, deprivation, and income | | A modified ﻿version of the global depression scale of HDL | | | |
| Lund and Cois, 2018 [20] | | Longitudinal | | | National representative | 2008-2012 | | | South Africa | | | The NIDS | | 11,440 individuals who were 15 years of age or older at the time of the first interview (2008) | | | | Material assets | | CESD-10 | | | |
| Martikainen et al., 2003 [33] | | Cross-sectional | | | City level | 1997/1999 | | | UK | | | The Whitehall II study | | 7,162 office staffs from London; aged 35–55 | | | | Income and wealth | | GHQ depression | | | |
| Mirowsky et al., 2001 [39] | | Longitudinal | | | National representative | 1995 and 1998 | | | USA | | | The ASOC | | 2,592 households, aged 18 or over | | | | Economic hardship | | CESD-7 | | | |
| Osafo et al., 2015 [58] | | Longitudinal | | | National representative | Income: 1992 and 2003; Wealth: 2004 and 2008 | | | UK | | | Income: the WLS; Wealth:  the ELSA. | | WLS: time1: 6,494 individuals & time 2: 4,812 individuals; ELSA: wave 1: 1,1264 individuals & wave 4: 6,425 individuals; Aged 50 and above. | | | | Income and wealth | | CESD | | | |
| Pool et al., 2017 [50] | | Longitudinal | | | National representative | 1992-2012 | | | USA | | | The HRS | | 19,281 late middle-aged adults, aged 51 to 64 years | | | | Negative wealth shock | | CESD-8 | | | |
| Pu et al., 2011 [51] | | Longitudinal | | | City level | 1996, 1999, 2003 | | | China | | | The SHLS | | 2,387 the middle age and elderly in Taiwan; aged 60 and above. | | | | Financial satisfaction | | CESD-10 | | | |

| **Study ID** | **Study type** | **Level of Study (national level or city level or town level or area level or others)** | **Years** | **Countries** | **Data source** | | **Population** | | **Exposures** | | **Depression measure** | |
| --- | --- | --- | --- | --- | --- | --- | --- | --- | --- | --- | --- | --- |
| Rautio et al., 2013 [35] | Cross-sectional | Hospital districts of Pirkanmaa, Southern Ostrobothnia and Central Finland | 2007 | Finland | | The FIN-D2D | 2,819 individuals; aged 45–74 years | Financial satisfaction | | BDI-21 | |  |
| Reeves et al., 2016 [38] | Repeated cross-sectional | National representative | 2009-2013 (4 waves) | UK | The APS | | 179,037 low-income persons aged between 16 and 69 years who were then renting housing in the private sector. | | Reductions in the Housing Benefit (HB) | | Self-reported depression | |
| Richardson et al., 2017 [59] | Longitudinal | University students/neighbourhood level | 2012-2014 | UK | A primary longitudinal study conducted on British first-year undergraduate students. | | 454 first-year British undergraduate students; aged between 17 and 57 | | Index of financial stress, debt stress, how to perceive student loan | | CESD-20 | |
| Ross and Huber, 1985 [52] | Cross-sectional | National representative | 1978 | USA | A telephone survey of a national probability sample of the U.S. households | | 680 wives and 680 husbands; aged 18-65 | | Economic hardship, family income, wife's and husband's earnings | | CESD-16 | |
| Sareen et al., 2011 [34] | Longitudinal | National representative | 2001/2002-2004/2005 (2 waves) | USA | ﻿The NESARC | | ﻿34,653 adults aged 20 and older | | Household income | | DSM-IV | |
| Sweet et al., 2013 [14] | Longitudinal | National representative | 2007/2008 | USA | The Add Health | | 8,400 respondents aged between 24 and 32 | | Financial debt amount, financial debt/asset, subjective relative debt | | Wave I: CESD-19; Wave III: CESD-9; Wave IV: CESD-5; | |
| Tran et al., 2018 [6] | Cross-sectional | University level/neighbourhood level | 2003 | USA | The NLSF | | 1,412 college students | | Student loan debt amount and debt stress | | CESD-13 | |

| **Study ID** | **Study type** | **Level of Study (national level or city level or town level or area level or others)** | **Years** | **Countries** | **Data source** | **Population** | **Exposures** | **Depression measure** |
| --- | --- | --- | --- | --- | --- | --- | --- | --- |
| Virtanen et al., 2008 [31] | Cross-sectional | National representative | 2000/2001 | Finland | The Health 2000 Study | 3,374 working population, aged 30-64 | Income | CIDI (DSM-IV) |
| Zimmerman and Katon, 2005 [16] | Longitudinal | National representative | 1979-2004 | USA | The NLSY | 8,489 young adults aged between 27 and 35 | Income | CESD-20 |
| Zurlo et al., 2014 [53] | Cross-sectional | Nationally representative | 2006 | USA | The HRS | 5,817 respondents; aged 50 and older | Occurrence of unsecured debt, Amount of unsecured debt | CESD-8 |
| N: No information was reported. | | | | | | | | |
