# Supplementary material for: Financial stress and depression in adults: A systematic review
Source: PLoS One. 2022 Feb 22;17(2):e0264041. doi: 10.1371/journal.pone.0264041 (PMC8863240; doi:10.1371/journal.pone.0264041)
Supplement: S2 Table — (DOCX) [file pone.0264041.s004.docx]

**S2 Table. Quality assessment form**

| **Study ID** | | **Country** | **Study design** | **Selection bias** | | **Withdrawals and drop-outs** | | **Confounding** | | **Data collection** | | **Data analysis** | | | **Reporting** | | **Overall rating** | |
| --- | --- | --- | --- | --- | --- | --- | --- | --- | --- | --- | --- | --- | --- | --- | --- | --- | --- | --- |
| Asebedo and Wilmarth, 2017 [41] | | USA | 2 | 1 | | N/A | | 2 | | 2 | | 1 | | | 1 | | 1 | |
| Alley et al., 2011 [40] | | USA | 2 | 1 | | N/A | | 1 | | 2 | | 1 | | | 1 | | 1 | |
| Berger et al., 2016 [26] | | USA | 2 | 1 | | N/A | | 1 | | 2 | | 1 | | | 1 | | 1 | |
| Boe et al, 2017 [42] | | Europe | 2 | 1 | | N/A | | 2 | | 2 | | 1 | | | 1 | | 1 | |
| Boey and Chiu, 2005 [36] | | China | 2 | 2 | | N/A | | 2 | | 2 | | 1 | | | 2 | | 2 | |
| Bridges and Disney, 2010 [13] | | UK | 2 | 1 | | 2 | | 1 | | 2 | | 1 | | | 1 | | 1 | |
| Butterworth et al., 2009 [15] | | South-east Australia | 2 | 1 | | 1 | | 2 | | 2 | | 1 | | | 1 | | 1 | |
| Butterworth et al., 2012 [30] | | Australian | 2 | 1 | | N/A | | 2 | | 2 | | 1 | | | 1 | | 1 | |
| Chen et al., 2016 [43] | | China | 2 | 1 | | N/A | | 1 | | 2 | | 1 | | | 1 | | 1 | |
| Cheung and Chou, 2017 [37] | | China | 2 | 1 | | N/A | | 2 | | 2 | | 1 | | | 1 | | 1 | |
| Chi and Chou, 2000 [44] | | China | 2 | 1 | | 1 | | 2 | | 1 | | 1 | | | 2 | | 1 | |
| Drentea and Reynolds, 2012 [54] | | USA | 2 | 1 | | 2 | | 1 | | 2 | | 3 | | | 1 | | 2 | |
| Drentea and Reynolds, 2015 [45] | | USA | 2 | 1 | | 2 | | 1 | | 2 | | 1 | | | 1 | | 1 | |
| Ellaway et al., 2016 [29] | | UK | 2 | 2 | | N/A | | 2 | | 2 | | 1 | | | 1 | | 2 | |
| Gathergood and John, 2012 [32] | | UK | 2 | 1 | | 3 | | 1 | | 2 | | 1 | | | 1 | | 2 | |
| Gillen et al., 2017 [27] | | USA | 2 | 1 | | N/A | | 1 | | 2 | | 1 | | | 1 | | 1 | |
| Hiilamo and Grundy, 2018 [28] | | Belgium, France, Germany | 2 | 1 | | N/A | | 1 | | 2 | | 1 | | | 1 | | 1 | |
| Hojman, 2016 [46] | | Chilean | 2 | 1 | | N/A | | 1 | | 2 | | 1 | | | 1 | | 1 | |
| Jo et al., 2011 [55] | Korea | | 2 | | 1 | | N/A | | 2 | | 3 | | 1 | 1 | | 2 | |  |

| **Study ID** | **Country** | **Study design** | **Selection bias** | **Withdrawals and drop-outs** | **Confounding** | **Data collection** | **Data analysis** | **Reporting** | **Overall rating** |
| --- | --- | --- | --- | --- | --- | --- | --- | --- | --- |
| Kim et al., 2016 [47] | Korea | 2 | 1 | N/A | 1 | 2 | 1 | 1 | 1 |
| Krause et al., 1991 [56] | USA, Japan | 2 | 1 | N/A | 2 | 2 | 1 | 2 | 2 |
| Krause et al., 1998 [57] | China | 2 | 3 | N/A | 2 | 2 | 1 | 2 | 2 |
| Leung and Lau, 2017 [48] | USA | 2 | 1 | N/A | 1 | 2 | 1 | 1 | 1 |
| Lorant et al., 2007 [49] | Belgian | 2 | 1 | N/A | 2 | 2 | 1 | 1 | 1 |
| Lund and Cois, 2018 [20] | South Africa | 2 | 1 | N/A | 2 | 2 | 1 | 1 | 1 |
| Martikainen et al., 2003 [33] | UK | 2 | 1 | N/A | 2 | 2 | 1 | 1 | 1 |
| Mirowsky et al., 2001 [39] | USA | 2 | 1 | N/A | 2 | 2 | 1 | 1 | 1 |
| Osafo et al., 2015 [58] | UK | 2 | 1 | 2 | 2 | 2 | 1 | 1 | 2 |
| Pool et al., 2017 [50] | USA | 2 | 1 | N/A | 1 | 2 | 1 | 1 | 1 |
| Pu et al., 2011 [51] | China | 2 | 1 | N/A | 2 | 2 | 1 | 1 | 1 |
| Rautio et al., 2013 [35] | Finland | 2 | 2 | N/A | 2 | 2 | 1 | 1 | 2 |
| Reeves et al., 2016 [38] | UK | 2 | 1 | N/A | 2 | 2 | 1 | 1 | 1 |
| Richardson et al., 2017 [59] | UK | 2 | 2 | N/A | 2 | 2 | 1 | 2 | 2 |
| Ross and Huber, 1985 [52] | USA | 2 | 1 | N/A | 2 | 1 | 1 | 1 | 1 |
| Sareen et al., 2011 [34] | USA | 2 | 1 | N/A | 2 | 2 | 1 | 1 | 1 |
| Sweet et al., 2013 [14] | USA | 2 | 1 | N/A | 1 | 2 | 1 | 1 | 1 |
| Tran et al., 2018 [6] | USA | 2 | 1 | N/A | 2 | 2 | 1 | 1 | 1 |
| Virtanen et al., 2008 [31] | Finland | 2 | 2 | N/A | 2 | 2 | 1 | 1 | 2 |
| Zimmerman and Katon, 2005 [16] | USA | 2 | 1 | N/A | 1 | 2 | 1 | 1 | 1 |
| Zurlo et al., 2014 [53] | USA | 2 | 1 | N/A | 2 | 2 | 1 | 1 | 1 |
