# Supplementary material for: Financial stress and depression in adults: A systematic review
Source: PLoS One. 2022 Feb 22;17(2):e0264041. doi: 10.1371/journal.pone.0264041 (PMC8863240; doi:10.1371/journal.pone.0264041)
Supplement: S3 Table — (DOCX) [file pone.0264041.s005.docx]

**S3 Table. Methods and findings extraction form**

| **Study ID** | **Econometrical methods** | **Subgroup analysis** | **Panel analysis** | **Exposures** | **Depression measure** | **Control for** | **Main findings** |
| --- | --- | --- | --- | --- | --- | --- | --- |
| Asebedo and Wilmarth, 2017 [41] | Ordinal logistic regression model | N | N | Financial strain and financial stress | CESD-8 | Objective financial stressors, health and socio-demographic characteristics | Financial strain is associated with reduced mental health. Lower levels of stress about ongoing financial strain reduces the negative relationship between financial strain and mental health. |
| Alley et al., 2011 [40] | Logistic regression model | N | N | Mortgage delinquency | CESD-8 | ﻿Age, gender, race/ ethnicity, marital status, smoking status, sources of debt, and annual household income, employment, health condition, physical activity level. | ﻿﻿Compared with nondelinquent participants, the mortgage-delinquent group were significantly more likely to develop incident depressive symptoms during follow-up. |
| Berger et al., 2016 [26] | Linear regression | N | Fixed effects estimation; Cross-lag structural equation estimation | Debt occurrence and debt amount; financial debt occurrence and financial debt amount; housing debt occurrence and housing debt amount | CESD-12 | Time-invariant baseline measures: male, race/ethnicity, the highest level of educational attainment by the respondent’s most educated parent Time-varying socioeconomic factors age, marital status, the proportion of household members, respondent education, the logarithm of total household income, working at the time of the interview; total household assets, current health status, and wave indicator. | Household debt is positively associated with greater depressive symptoms. However, this association appears to be driven by short-term (unsecured) debt; little evidence of associations with depressive symptoms for mid- or long-term debt was found. |
| Boe et al, 2017 [42] | Multi-level OLS regression. | Age | N | Financial difficulties in childhood | CESD-8 | Age, gender, education, family conflict, highest education in family, Marital status, and social meetings | Financial difficulties in childhood was an influential predictor of depression scores in younger adults for many of the investigated countries. |
| Boey and Chiu, 2005 [36] | Hierarchical multiple regression model | Gender | N | Financial status: income and perceived income sufficiency | GDS-15 | Age, education, marital status, physical health | Perceived financial sufficiency was significantly associated with reduced depressive symptoms in both genders. The relationship between objective income and depressive symptoms was not significant. |

| **Study ID** | **Econometrical methods** | **Subgroup analysis** | **Panel analysis** | **Exposures** | **Depression measure** | **Control for** | **Main findings** |
| --- | --- | --- | --- | --- | --- | --- | --- |
| Bridges and Disney, 2010 [13] | Probit regression & Logistic regression | N | Fixed-effect logistic regression | Objective debt variables, subjective debt problem, subjective financial stress | Self-reported depression | Age, gender, marital status and the number of children, educational qualifications and employment status and current health status | There is a positive association between the probability of reporting depression and self-reported problems of indebtedness and financial stress. Only a weak link exists between objective measures of the financial position of the household and psychological stress. |
| Butterworth et al., 2009 [15] | Logistic regression model | Age (young, middle, old) | N | Financial hardship in wave 2, and subjective financial difficulty in wave 1 | GDS-9 | Age, gender, marital status, and presence of dependent children (aged under 15) in the household, physical disability, labour-force status, educational attainment, housing tenure, and childhood adverse financial situation | Current financial hardship was strongly and independently associated with depression above the effects of other measures of social and economic position and demographic characteristics. There was no significant difference in the association between hardship and depression across different age groups. |
| Butterworth et al., 2012 [30] | Logistic regression model & Multinomial logistic regression | Age (young, middle, old) | N | Household income and financial hardship | ICD-10 | Age, sex, partner status, location | Financial hardship was strongly associated with depression while the association between household income and depression was not significant. ﻿Hardship was more strongly associated with current depression than with prior history of depression. The relative effect of hardship was strongest in late adulthood, but the absolute effect of hardship was greatest in middle age. |
| Chen et al., 2016 [43] | Linear regression | Age (45-60 & over 60); income level; educational level; mental health status | N | Pension enrolment and pension income | CESD-20 | Age, gender, ethnicity, CCP membership, married or not, year of education, NCMS, chronic disease, total assets, migration ratio, as well as several regional characteristics. | Pension enrolment and pension income significantly decreased depressive symptoms of pensioners aged 60 and above. ﻿This effect was more pronounced among individuals eligible to claim pension income, among populations with more financial constraints, and among those with worse baseline mental health. |

| **Study ID** | **Econometrical methods** | **Subgroup analysis** | **Panel analysis** | **Exposures** | **Depression measure** | **Control for** | **Main findings** |
| --- | --- | --- | --- | --- | --- | --- | --- |
| Cheung and Chou, 2017 [37] | Logistic regression model | N | N | Income poverty and material deprivation | GDS-15 | Age, gender, marital status, education level, financial strain, self-rated health, functional disability, poor sleeping quality, protective factors such as social support, engagement in cultural activities a neighbourhood collective efficacy | The association between income and depressive symptoms disappeared once material deprivation was controlled for. When all the covariates were entered, the association of material deprivation with depressive symptoms remained significant but was slightly weaker. |
| Chi and Chou, 2000 [44] | Multiple regression | Gender | N | Financial strain | CESD-20 | Age, gender, marital status, years of education, physical health, social support | The elderly persons with higher financial strain reported more depressive symptoms three years later. Financial strain influenced depression equally among men and women. |
| Drentea and Reynolds, 2012 [54] | Logistic regression model | Assets level | N | Occurrence of debt, credit card debt, and debt stress | CESD-20 | Income, assets, and homeownership/home value, education, age, race, gender, employment status, health insurance coverage, marital status, physical disability status, children in the household, and prior depression, prior anxiety, prior anger, and health problems up to 25 | ﻿Being in debt was associated with higher depressive symptomatology, controlling for all the variables in the left cell. ﻿The effect of debt on mental health did not vary by level of assets. |
| Drentea and Reynolds, 2015 [45] | Multivariate Generalised Least Squares Regression | N | N | Occurrence of debt and economic hardship | CESD-20 | Education, age, race, gender, employment status, health insurance coverage, marital status, physical disability status, presence of dependent children in the household, and prior mental and physical health problems, earlier health problems, the lagged value of the dependent variable as assessed in the first wave of the study, income, assets, and homeownership/home value | Both debt and economic hardship are associated with negative mental health, and the effect of economic hardship more consequential than debts. The influence of hardship is partly mediated by mastery. This is not true of debt. |

| **Study ID** | **Econometrical methods** | **Subgroup analysis** | **Panel analysis** | **Exposures** | **Depression measure** | **Control for** | **Main findings** |
| --- | --- | --- | --- | --- | --- | --- | --- |
| Ellaway et al., 2016 [29] | Generalised linear model & Logistic regression | N | N | Material assets | HADS depression. | Age, sex, marital status and income or social class | Housing tenure and car ownership are associated with mental health, after taking known correlates (age, sex, social class, income) into account. |
| Gathergood and John, 2012 [32] | Linear regression | N | Fixed-effect linear regression; IV least squares regression | The occurrence of problem debt and debt stress | GHQ-12 depression | Age, gender, marital status, has children, ethnicity, homeowner, regional dummies, education and employment, household income | There is a causal effect of the perceived problem debt on the worsening of psychological health at the household level. There is a sizable social norm effect in the perceived debt-mental health relationship. |
| Gillen et al., 2017 [27] | OLS regression model | N | N | Debt occurrence, financial assistance from family, public assistance | CESD-8 | Age, education, race, marital status, health status, education, gender, employment status, income, and wealth | (As an aggregated measure, financial constraints were positively associated with depression in mid-age and in late life). Having any type of debt (including financial debt and mortgage loan) was positively associated with more depressive symptoms.  The relationship between the occurrence of debt and depressive symptoms was partially accounted for by perceived control over one’s financial circumstances. |
| Hiilamo and Grundy, 2018 [28] | Linear mixed model with random intercepts | Country and gender | Linear mixed model with random intercepts | Financial debt amount and housing debt amount | CESD-12 | Education level, employment status, homemaker for women, household gross wealth, physical health status, age (age and age-squared), marital status, housing status, household size, time and country × time interaction dummies. | After adjustment, low or substantial financial debt was associated with a higher number of depressive symptoms in all countries. Housing debt was strongly linked to depressive symptoms for women while the association was weaker for men. Associations between financial debt and depression were also evident in analyses of within-individual changes in depressive symptoms for a longitudinal sub-group. |

| **Study ID** | **Econometrical methods** | **Subgroup analysis** | **Panel analysis** | **Causal effect** | **Depression measure** | **Control for** | **Main findings** |
| --- | --- | --- | --- | --- | --- | --- | --- |
| Hojman, 2016 [46] | OLS regression model | N | N | N | CESD-8 | Socio demographics, family characteristics, health problems, personality traits, assets, personal history, health risk factors, regional dummies | Depressive symptoms are higher for those who persistently have a high debt level, followed by those who transit from moderate to high debt level. Those who transition from over-indebtedness to moderate debt levels have no additional depressive symptoms compared to those with trajectories of moderate debt throughout (never over-indebted). The association between debt and depressive symptoms seems to be driven by non-mortgage debt -primarily consumer credit- or late mortgage payments; secured debt (secured by collateral) per se is not associated with depressive symptoms. |
| Jo et al., 2011 [55] | Multiple logistic regression model | Gender & Income level | N | N | CESD-20 | Gender, age, educational status, marital status. | An increase by a million won/month of income significantly reduced the possibility of depressive symptoms. Among males, only the highest average monthly family income showed such a tendency; among females, a higher income level was associated with a lower possibility of depressive symptoms for all income groups. |
| Kim et al., 2016 [47] | Hierarchical logistic regression model | Education | N | N | CESD-11 | Age, gender, education level, marital status, employment status, family satisfaction level, perceived health status, and chronic disease status | Low current economic status was associated with a higher likelihood of depressive symptoms. In particular, the low current-low childhood economic status group showed the highest likelihood of depressive symptoms, suggesting the adverse mental health effects of prolonged poverty. |

| **Study ID** | **Econometrical methods** | **Subgroup analysis** | **Panel analysis** | **Exposures** | **Depression measure** | **Control for** | **Main findings** |
| --- | --- | --- | --- | --- | --- | --- | --- |
| Krause et al., 1991 [56] | Latent variable model | N | N | Financial strain | CESD-6 | Age, sex, education | Financial strain tends to erode feelings of control and self-worth in both the US and Japan, and the weakening of these personal resources tends to increase depressive symptoms. |
| Krause et al., 1998 [57] | Hierarchical OLS regression model | N | N | Financial strain, received economic support, anticipated economic support. | CESD-6 | Age, sex, education, marital status, urban residence or not, occupational status. | There is a strong relationship between ongoing financial problems and depression scores for Chinese older people. The relationship between financial strain and depressive symptoms becomes larger as older adults in China receives more economic support. The relationship becomes weaker as the level of anticipated support increases. |
| Leung and Lau, 2017 [48] | Linear regression | Mortgagors vs All homeowners | Fixed-effect estimation & First difference estimation & Fixed effect Instrumental variable estimation & First difference instrumental variable estimation | Mortgage loan to house value | CESD-8 | Age, marital status, annual average unemployment rates in the county of residence, year dummies to capture time trends and state dummies to capture persistent health differences across states | Results from pooled OLS estimations show that excessive mortgage indebtedness is associated with a greater number of depressive symptoms. After adjusting for individual-level heterogeneity using panel estimation, these associations become statistically insignificantly different from zero. Using state-level home prices as an instrument variable in a panel IV framework, results show that Panel IV estimations suggest that having a high mortgage loan to home value (LTV), defined as LTV at or above 80 %, leads to more depressive symptoms. |

| **Study ID** | **Econometrical methods** | **Subgroup analysis** | **Panel analysis** | **Exposures** | **Depression measure** | **Control variables** | **Main findings** |
| --- | --- | --- | --- | --- | --- | --- | --- |
| Lorant et al., 2007 [49] | ﻿Conditional logistic regression | N | Fixed effect estimation | Subjective financial strain, poverty, deprivation, and income | A modified ﻿version of the global depression scale of HDL | Age, gender, education, employment, civic participation, living with a partner or spouse | ﻿  1-year increases in material  hardship such as financial strain, deprivation and poverty led to an increase in depressive symptoms, ﻿  and caseness ﻿of major depression. |
| Lund and Cois, 2018 [20] | Weighted linear regression | N | Cross-lagged structural equation model | Material assets | CESD-10 | Gender, race, age and categorical education | Worse individual economic status at time 1 and 2 were independently associated with worse depression two years later at time 2 respectively. Conversely worse depression at time 1 and time 2 was independently associated with worse economic status at time 2 respectively. In addition, the "effect" of depression on future assets was stronger among people with fewer baseline assets. |
| Martikainen et al., 2003 [33] | Logistic regression | Gender | N | Income and wealth | GHQ depression | Age, health at baseline, employment status, number of economically active adults, number of children and number of all persons in the household, marital status, education, and employment grade | The association between income (especially individual income) and depression can be largely accounted for by health status at baseline and other socioeconomic factors. There is a strong association between household wealth and depression. |
| Mirowsky et al., 2001 [39] | Linear regression | Age & income level | Subsequent and concurrent change model | Economic hardship | CESD-7 | Age, sex, race, education, marital status, household income, non-wage household, chronic conditions | Economic hardship is statistically significantly associated with depression in all age groups, and the amount of the relationship is decreases with older age, both cross-sectional and over time. |

| **Study ID** | **Econometrical methods** | **Subgroup analysis** | **Panel analysis** | **Exposures** | **Depression measure** | **Control for** | **Main findings** |
| --- | --- | --- | --- | --- | --- | --- | --- |
| Osafo et al., 2015 [58] | Linear regression | N | N | Income and wealth | CESD | Income-depression: gender, age, age squared, household size, level of highest education since high school, marital status, current employment status, and retirement. Wealth-depression: gender, age, age squared, household size, employment status, marital status, educational attainment, tenure, retirement status was used | It is the rank of a person’s income or wealth within a social comparison group, rather than income or wealth themselves or their deviations from the mean within a reference group, that is more strongly associated with depressive symptoms. |
| Pool et al., 2017 [50] | Logistic regression model | N | Nested cross-over model within individuals with conditional logistic regression | Negative wealth shock | CESD-8 | Cross-over model control for: Time-varying sociodemographic covariates (including marital status, household annual income, current unemployment status, and health insurance status). Panel regression control for both time-varying and Time invariant variables (including gender, race/ethnicity, age and educational attainment were assessed at baseline). | Negative wealth shock caused the increased risk of more severe depressive symptoms amongst late middle-aged people. |
| Pu et al., 2011 [51] | Generalised estimation equation (GEE) | Education level | N | Financial satisfaction | CESD-10 | Baseline age, sex, marital status, educational level, ethnicity, smoking, and number of people living together | Among those who were dissatisfied with their financial position, those who were illiterate had an odds ratio of 8.3% for having depressive symptoms compared with those who were very satisfied with their financial position. |
| Rautio et al., 2013 [35] | Logistic regression model | N | N | Financial satisfaction | BDI-21 | Gender, age, marital status, number of chronic diseases, smoking, binge drinking, physical activity, education, and household income | Individuals who were less satisfied with their financial situation were more likely to suffer from depressive symptoms even after controlling for all the variables in the left cell. |

| **Study ID** | **Econometrical methods** | **Subgroup analysis** | **Panel analysis** | **Exposures** | **Depression measure** | **Control for** | **Main findings** |
| --- | --- | --- | --- | --- | --- | --- | --- |
| Reeves et al., 2016 [38] | Linear probability model | N | N | Reductions in the Housing Benefit (HB) | Self-reported depression | Age, sex, employment status, geographical region, ethnicity, number of dependent children in the household under the age of 19 years, income, occupation, education, whether the respondent was a job-seeker s allowance claimant, and the date of interview | Reducing housing support to low-income persons in the private rental sector increased the prevalence of depressive symptoms in the UK. |
| Richardson et al., 2017 [59] | Hierarchical linear multiple regression model | N | N | Index of financial stress, debt stress, how to perceive student loan | CESD-20 | Age, gender, disability, mature student, ethnicity, family affluence scale, and symptoms at baseline | Greater financial difficulties such as being unable to pay the bills predicted greater depression and stress cross-sectionally. Depression worsened over time for those who had considered abandoning studies or not coming to the university for financial reasons, and there were effects on how students viewed their student loans. Greater stress about debt predicted greater depression when examined cross-sectionally. |
| Ross and Huber, 1985 [52] | Structural equation model with MLR estimation | Gender subgroup | N | Economic hardship, family income, wife's and husband's earnings | CESD-16 | Age, education, race, number of young children, earnings, family income | Economic hardship increases both women/men's depression levels. |

| **Study ID** | **Econometrical methods** | **Subgroup analysis** | **Panel analysis** | **Exposures** | **Depression measure** | **Control for** | **Main findings** |
| --- | --- | --- | --- | --- | --- | --- | --- |
| Sareen et al., 2011 [34] | ﻿Multiple logistic regression model | Age, sex | N | Household income | DSM-IV | ﻿Age, sex, marital status, race and number of persons in the household. | ﻿Participants with household income of less than $20000 per year were at increased risk of incident mood disorders during the 3-year follow-up period in comparison with those with income of $70 000 or more per year. A decrease in household income during the 2 time points was also associated with an increased risk of incident mood, anxiety, or substance use disorders (adjusted odds ratio, 1.30; 99% confidence interval, 1.06-1.60) in comparison with respondents with no change in income. The baseline presence of mental disorders did not increase the risk of change in personal or household income in the follow-up period. |
| Sweet et al., 2013 [14] | OLS multiple regression model | N | N | Financial debt amount, financial debt/asset, subjective relative debt | Wave I: CESD-19; Wave III: CESD-9; Wave IV: CESD-5; | Wave I and Wave III socio-economic, psychological, and health factors (general health, depressive symptoms, medical exams, health insurance, skipped medical care, disease diagnoses, hospitalizations, exercise, smoking, diet, BMI, income, education, parental education, homeownership, and race/ethnicity); Wave IV socioeconomic and demographic factors (number of people in the household, education, income, smoking, physical activity, marital status, health insurance, job loss, and homeownership) | High calculated financial debt to assets ratio and subjective debt to assets ratio are independently associated with higher depression. The effect remains significant after controlling for prior socioeconomic status, psychological and physical health, and other demographic factors. High financial debt amount was an inconsistent predictor of more depressive symptoms. |

| **Study ID** | **Econometrical methods** | **Subgroup analysis** | **Panel analysis** | **Exposures** | **Depression measure** | **Control for** | **Main findings** |
| --- | --- | --- | --- | --- | --- | --- | --- |
| Tran et al., 2018 [6] | Structural equation model with MLR estimation | N | N | Student loan debt amount and debt stress | CESD-13 | Household income, U.S. born, gender, mother's highest level of education, and father's highest level of education | Student loan debt was positively linked to debt stress, and debt stress was linked to poorer general health and greater depressive symptomatology. |
| Virtanen et al., 2008 [31] | Logistic regression model | Gender & Income level | N | Income | CIDI (DSM-IV) | Age, non-working factors (marital status, housing conditions, lack of social support outside work, victim of violence, smoking, abnormal somatic health, number of physical symptoms) and working factors (job demands, lack of job control, lack of social support at work, lack of educational prospects at work, job insecurity). | Low income is associated with frequent mental disorders among a working population. Work factors among men and non-work factors among women contribute to the income differences in mental health. |
| Zimmerman and Katon, 2005 [16] | Negative binomial regression  model | Gender & Income level | Fixed effect regression & Instrumental variable negative binomial hurdle regression | Income | CESD-20 | Non-economic correlates: age (and age-squared), race/ ethnicity, region, urbanity, marital status, the Rosenberg self-esteem scale, self-report of any major current or past physical health problems, and whether there are any children under 12 in the respondent’s household. Economic covariates: current employment status, the logged ratio of debts-to-assets, logged years of education completed, job type, insurance status, and whether the respondent owns their own home. | Income is significantly correlated with depression after controlling for non-economic correlates. Statistical strength and the magnitude of the association between income and depressive symptoms are less with other economic covariates controlled. The association between depression and income has a greater magnitude at lower income levels than at higher ones. |

| **Study ID** | **Econometrical methods** | **Subgroup analysis** | **Panel analysis** | **Exposures** | **Depression measure** | **Control for** | **Main findings** |
| --- | --- | --- | --- | --- | --- | --- | --- |
| Zurlo et al., 2014 [53] | OLS regression model | N | N | Occurrence of unsecured debt, Amount of unsecured debt | CESD-8 | Age, education, gender, marital status, race, employment status, self-reported health, household income, household net worth, ratio of housing costs/income | Both the occurrence of unsecured debt and the amount of unsecured debt was positively and significantly associated with more depressive symptoms independently. The negative effects of unsecured debt on depressive symptoms are largely accounted for by perceived control over personal financial circumstances. |

N: No information reported.
