# Supplementary material for: Financial stress and depression in adults: A systematic review
Source: PLoS One. 2022 Feb 22;17(2):e0264041. doi: 10.1371/journal.pone.0264041 (PMC8863240; doi:10.1371/journal.pone.0264041)
Supplement: S1 Appendix — (DOCX) [file pone.0264041.s006.docx]

**S1 Appendix. Sample search in PsycINFO**

Database: PsycINFO as an example

Search Strategy:

**Financial variables**

1. income.mp.
2. (debt* or indebt* or loan* or mortgage*).mp.
3. wealth.mp.
4. asset*.mp.
5. financ*.mp.
6. (economic situation* or economic stat* or economic condition* or economic position* or economic hardship* or economic str* or economic difficul*).mp.
7. (financial situation* or financial stat* or financial condition* or financial position* or financial str* or financial hardship* or financial satisf* or financial difficult*).mp.
8. (poverty or deprivation).mp.
9. 1 or 2 or 3 or 4 or 5 or 6 or 7 or 8

**Individual and/or household**

1. (household* or family). mp.
2. (individual* or personal).mp.
3. 10 or 11

**Depression**

1. depress*.mp.
2. depressive disorder*.mp.
3. depressive symptom*.mp.
4. depressed mood*.mp.
5. mood disorder*.mp.
6. affective disorder*.mp.
7. dysthymia*.mp.
8. 13 or 14 or 15 or 16 or 17 or 18 or 19

**Study types**

1. (observational or cross-sectional or longitudinal or cohort or panel or retrospective or prospective or survey or questionnaire).mp.

**The combination of all terms**

1. 9 and 12 and 20 and 21
2. limit 22 to English
