# Supplementary material for: Financial stress and depression in adults: A systematic review
Source: PLoS One. 2022 Feb 22;17(2):e0264041. doi: 10.1371/journal.pone.0264041 (PMC8863240; doi:10.1371/journal.pone.0264041)
Supplement: S2 Appendix — (DOCX) [file pone.0264041.s007.docx]

**S2 Appendix. Data extraction form**

**A. Characteristics of studies**

1. Title

2. Year

3. Authors

5. Journal name

6. Aim of the study

7. Countries

**B. Data collection information**

1. Study type

2. Did the study use primary or secondary data?

3. Data source and dates

4. Response Rate

5. Was information for non-responders described?

6. Level of Study (national level or city level or town level or area level or others)

7. Eligibility criteria for participants?

8. Ethical approval

**C. Population description**

1. Sample Size

2. Age group

3. Mean/Median age of the participants

4. Gender structure

**D. Exposures**

1. Any exposures related to household/personal finances

2. Objective measures or subjective measures

3. The measure of related exposures

4. Exposure’s categories

5. Definition of exposures

6. The validity of the measure of exposures (Cronbach’s alpha or correlation coefficient etc)

**E. Outcomes**

1. Study outcomes related to depressive symptoms/ depression/depressive disorder)

2. Measures of depression

3. Definition of depression

4. Cut-off points/ thresholds of depression if applicable

5. The validity of the measure of outcome (Cronbach’s alpha or correlation coefficient etc.)

**F. Data and analyses**

1. Statistical methods

2. Confounders controlled

3. Adjust for reverse causality or not? And how did the authors deal with it?

4. Subgroup analyses

5. Robustness check

**G. Main results (household finances and depress* relationships only)**

1. Unadjusted results

2. Results after adjusting for confounders if applicable

3. Results after adjusting for reverse causality if applicable

**I. Notes**
